# Significant effects of negligible amount of H_2_O_2_ on photocatalytic efficiency of MIL-125 and NH_2_-MIL-125 nanostructures in degradation of methylene blue†

**DOI:** 10.1039/d4ra05733c

**Published:** 2024-09-23

**Authors:** Afsaneh Mahmoodi, Davoud Dorranian, Hamed Abbasi

**Affiliations:** a Plasma Physics Research Center, Science and Research Branch, Islamic Azad University Tehran Iran doran@srbiau.ac.ir; b Department of Imaging Physics, Faculty of Applied Sciences, Delft University of Technology Delft The Netherlands h.abbasi@tudelft.nl; c Center for Optical Diagnostics and Therapy, Department of Otorhinolaryngology and Head and Neck Surgery, Erasmus MC, University Medical Center Rotterdam 3015 CN Rotterdam The Netherlands

## Abstract

The notable impact of a trace amount of hydrogen peroxide (H_2_O_2_) on the photocatalytic performance of Ti-based metal–organic frameworks (MOFs), namely MIL-125 and NH_2_-MIL-125, in the purification of water polluted with chemical agents was studied experimentally. MIL-125 and NH_2_-MIL-125 were synthesized using the solvothermal method and were characterized by a variety of diagnostic methods. NH_2_-MIL-125 exhibited a bandgap of 2.8 eV compared to 3.65 eV for MIL-125 with optimal visible light capture capability, indicating the outstanding photodegradation activity of the synthesized MOFs. In addition, the photocatalytic performance of MIL-125 and NH_2_-MIL-125 was tested for the degradation of methylene blue (MB) as a chemical pollutant in water under both dark conditions and irradiation by visible light and a UVC lamp. NH_2_-MIL-125 exhibited a significantly higher photodegradation rate compared to MIL-125 due to the presence of the amino group, higher surface electronegativity and slightly lower bandgap. Furthermore, the effect of H_2_O_2_ as an electron acceptor on the efficiency of MB degradation was investigated, which markedly enhanced the photocatalytic MB degradation performance due to the ligand-to-metal charge transfer mechanism, particularly for NH_2_-MIL-125, under all tested conditions.

## Abstract 2

The notable impact of a trace amount of hydrogen peroxide (H_2_O_2_) on the photocatalytic performance of Ti-based metal–organic frameworks (MOFs), in the purification of water polluted with chemical agents was studied experimentally.

## Graphical abstract

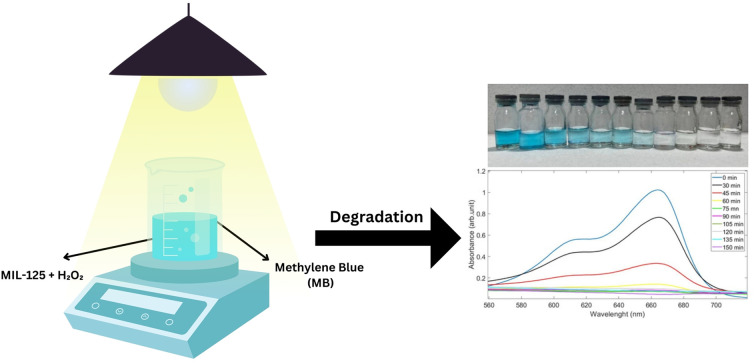

## 1. Introduction

The demand for drinking water has increased in recent years, and the availability of purified water has become a major concern. Thus, different purification technologies are being sought in water and wastewater treatment, especially to purify some new groups of contaminants that are not readily removed by conventional technologies. Various industries can contaminate water with dyes, including the textile, pharmaceutical, food, cosmetic, plastic, medicine, and leather industries. These industries, known for their substantial water consumption and environmental impact during the production processes, are major contributors to wastewater pollution. These pollutants, including heavy metals, polyenes, and dyes, pose significant environmental concerns and are categorized as a severe problem. They are highly toxic, leading to various health issues in humans, including excessive sweating, cognitive impairment, and methemoglobinemia.^1–3^ It has been reported that in the textile industry, wastewater accounts for approximately 80% of the total discharge, emphasizing the global significance of addressing dye removal from wastewater.^4^ As a consequence, more than 15% of the global population lacks access to safe drinking water, which is a fundamental need for human society.^5^

Common methods for removing dyes include adsorption, flocculation, coagulation, and degradation.^6–8^ While adsorption is an uncomplicated and easy-to-use technique, it requires proper disposal of the adsorbed organic contaminants. In recent years, researchers have mainly focused on advanced oxidation processes, like photocatalysis, to eliminate organics from water because they can degrade and mineralize these pollutants.^9^ The organic dye pollutants, such as methylene blue (MB), interrupt the reoxygenation of aquatic systems and increase toxicity for humans and the environment.^10^ MB is widely utilized for various applications, including dyeing cotton, wool and silk.^11^ It is also widely used as a contrast agent in fluorescence-guided surgery^12^ and, thus, may be present in different wastewaters, including the textile industry and hospital wastewater.

Furthermore, hydrogen peroxide (H_2_O_2_) as an electron acceptor can significantly boost photocatalytic degradation. Easy preparation, impressive photocatalytic performance, and strong stability render them excellent candidates for decolorizing organic pollutants in wastewater under visible light. H_2_O_2_ can enhance photocatalysis by acting as a sacrificial electron donor. In the presence of light and a photocatalyst, H_2_O_2_ can undergo photolysis, producing reactive oxygen species (ROS) like hydroxyl radicals. These ROSs can then participate in various oxidation reactions, leading to more efficient degradation of pollutants or activation of catalytic processes. The practical way to increase the photodegradation of MB is to add a strong oxidant.^13^ H_2_O_2_ increases the formation rate of hydroxyl radicals and enhances the degradation of compounds at low concentrations. This is due to the efficient generation of OH and inhibition of electron–hole pair recombination, as H_2_O_2_ is an electron acceptor.^14,15^ H_2_O_2_ is considered one of the most potent oxidizing potential catalysts for photocatalysis and has garnered significant attention for its potential in environmental remediation and energy conversion.

Metal–organic frameworks (MOFs) are a recently emerged porous material that has experienced rapid development over the last ten years and can be used as photocatalysts. With their benefits of a substantial specific surface area and customizable pore diameter/characters, MOFs hold promise for applications in gas storage, molecular separation, and adsorption.^16–18^ MOFs are a group of very promising porous crystalline inorganic–organic hybrid materials that have become one of the fastest-growing fields in both materials' science and chemistry in the last two decades.^19^ MOFs have shown great advantages for photocatalysis as a result of their flexible structure design and unique physiochemical properties compared with traditional photocatalysts.^20–22^ Researchers have shown that the photocatalytic presentation of MOF catalysts can be amplified by combination with semiconductors as a linker or metal center.^23^ Among MOFs, scientists have become interested in Ti-based MOFs, such as MIL-125 (Material of Institute Lavoisier-125, titanium 1,4-benzenedicarboxylate) and its amino-modified variant, NH_2_-MIL-125 (titanium 2-amino-1,4-benzenedicarboxylate) because of their unique properties such as high stability, catalyst activity, photocatalytic properties, biocompatibility and versatility. MIL-125 has been reported in 2009 as the first crystalline porous carboxylate-based Ti-MOF.^24^ Various strategies have since been attempted to exploit its photocatalytic potential. Among them, ligand tuning has been considered as a feasible and efficient strategy. For instance, if we consider the organic ligand of MIL-125, terephthalic acid (H_2_BDC), and introduce an amine group through grafting, leading to the formation of 2-amino terephthalic acid (NH_2_BDC), we can generate an isostructural MOF known as NH_2_-MIL-125. The presence of the amine group within the organic ligand acts as a chromophore, resulting in the narrowing of the bandgap from approximately 3.7 eV in MIL-125 to about 2.7 eV in NH_2_-MIL-125; the main contribution to the change in optical absorption is the dielectric confinement effect which is size dependent and related to the number density of particles.^25–30^ This narrowing enables the utilization of visible light. MIL-125 is responsive to light in the UV range, but NH_2_-MIL-125 can utilize visible light, expanding its potential applications in photocatalysis under visible light conditions. In this paper, we discuss comparing these two MOFs, as most research has focused on the visible light performance of NH_2_-MIL-125.

## 2. Experimental

### 2.1. Material

The following materials were obtained from Merc, Germany: 2-amino terephthalic acid (NH_2_BDC) (ATA, 99%), terephthalic acid (H_2_BDC), *N*,*N*-dimethylformamide (DMF, ≥99.8%), MeOH (methanol) (99.8%). Titanium(iv) isopropoxide (TTIP) (≥97%) was obtained from Sigma Aldrich Chemicals. All chemicals were used as received without further purification, and distilled water was utilized for the whole experiment.

### 2.2. Synthesis of MIL-125

The procedure was adapted from ref. 24. In a single preparation, 1 g (6 mmol) of H_2_BDC was first dissolved into a mixture of 2 mL methanol and 18 mL DMF. Afterwards, 0.568 mL TTIP (2 mmol) was added to the solution under stirring. The mixture was then sonicated for 20 min and transferred into an autoclave, reacting at 150 °C for 18 h. The resulting powders were collected *via* centrifugation (5000 rpm, 5 min) and washed with DMF and MeOH three times each. The products were then dried overnight in the oven at 150 °C. This sample is named as MIL-125.

### 2.3. Synthesis of NH_2_-MIL-125

**Fig. 1:**
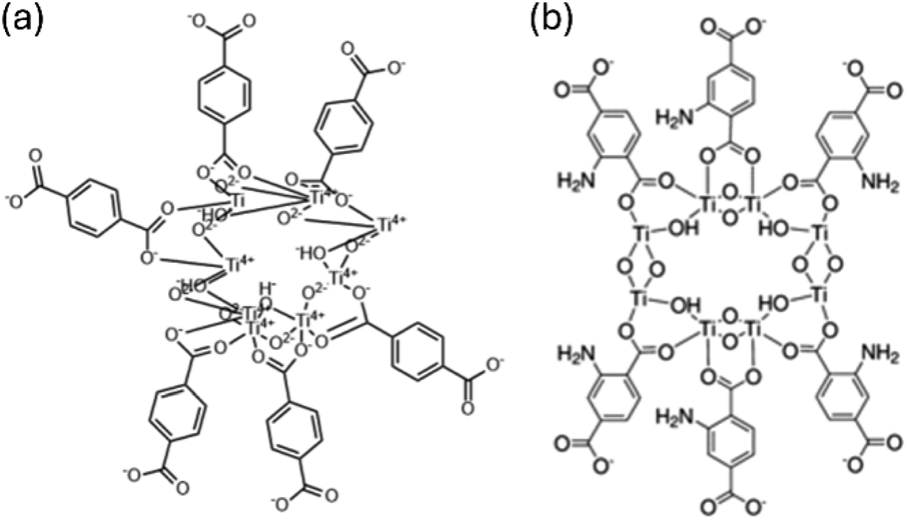
The molecular structures of (a) MIL-125 and (b) NH_2_-MIL-125.

The procedure was adapted from ref. 31. In a single synthesis, 40 mL of anhydrous DMF and 10 mL of HPLC MeOH were mixed as solvents, then 568.1 mg of NH_2_BDC was added to the mixture and stirred at room temperature till the yellow organic linker was fully dissolved. Then, 0.568 mL TTIP was added at the end, followed by stirring for another 3 min. The resulting solution was solve-thermally reacted in a 100 mL autoclave at 150 °C for 24 h. The resulting powders were collected *via* centrifugation (5000 rpm, 5 min) and washed with DMF and MeOH three times each. The products were then dried overnight in the oven at 150 °C. This sample is named as NH_2_-MIL-125. The molecular structures of these two types of MOFs are presented in Fig. 1.

### 2.4. Characterization methods

MIL-125 and NH_2_-MIL-125 were thoroughly characterized. The surface morphology was identified by a Field Emission Scanning Electron Microscope (FESEM). FESEM was conducted using the ZEISS Sigma VP model. Fourier-transform infrared spectroscopy (FTIR) was conducted using Thermo Nicolet Nexus 860 to investigate the chemical composition of the synthesized MOFs, and energy dispersive X-ray analysis (EDAX) was investigated using a detector from Oxford instruments. X-ray diffractometer (XRD-MAC Science M03XHF) was used to distinguish the crystal phase, and its patterns in this work were collected with X'Pert Pro multi-purpose diffractometer. Photoluminescence (PL) emission spectra were measured with the Cary Eclipse model from Varian equipped with a Xenon lamp and 345 nm excitation wavelength for MIL-125 and 429 nm excitation for NH_2_-MIL-125. Light absorption properties were characterized by PG instrument T92+ model UV-vis spectrophotometer, and bandgap (*E*_g_) was determined by Tauc plot from absorbance.

### 2.5. Photocatalytic degradation of MB

The photocatalytic activities of MIL-125 and NH_2_-MIL-125 were evaluated by the photodegradation of MB dye under dark, UVC lamp (the light source used was a 110 W Osram Hg lamp contains mercury, which can provide UVC light) and visible light (the light source used was a 300 W blue LEDs, which can provide visible light) irradiation in open air and at room temperature. The distance between the light source and the beaker containing the reaction mixture was fixed at 10 cm. The initial concertation of MB was prepared at 5 ppm in deionized water. The MOFs-based photocatalysts (5.00 mg) were dispersed into a 100.0 mL MB solution, and 1 mL H_2_O_2_ was added in a 150 mL beaker. Before irradiation, the suspension was magnetically stirred for 30 min in the dark to investigate the adsorption properties of MB onto the photocatalyst surface. The lights were switched on after reaching the adsorption–desorption equilibrium to initiate the photodegradation study. The suspensions were stirred for 120 minutes. Subsequently, 4 mL of MB solution was withdrawn at 0, 30, 45, 60, 75, 90, 105, 120, 135 and 150 minutes of time intervals. The photocatalytic activities of MIL-125 and NH_2_-MIL-125 were monitored from the variation of the color in the reaction system and measuring the maximum absorbance intensity of the MB chromophoric group at *λ*_max_ = 664 nm.

To have a better understanding of the reaction, the kinetics of the MB degradation catalysis by two photocatalysts, the kinetic constants of the reaction rate were determined according to the pseudo-first-order kinetic model as follows

1 ln(*C*/*C*_0_) = −*kt*

Where *t* is the irradiation time, *k* is the kinetic constant, *C*_0_ is the initial concentration (ppm), and *C* is the MB (ppm) concentration at a certain irradiation time.

## 3. Result and discussion

### 3.1. Characterization

The mechanism of photocatalysis of MB is irradiation by photons with energy equal to or greater than the bandgaps of MOFs. Electrons (e^−^) will be excited from the valence band (VB) to the conduction (CB) band, leaving holes (h^+^) in the valence band (eqn (2) and (3)). The photo-excited holes have a strong oxidant ability and can directly oxidize adsorbed organic molecules or react with hydroxyl ions (OH^−^) to generate hydroxyl radicals (·OH) (eqn (4)). The formed ·OH radicals possess a strong oxidation capacity and can also oxidize the surface adsorbed organic molecules. Meanwhile, photoexcited electrons can be trapped by molecular oxygen to form superoxide radicals (·O_2_^−^) (eqn (5)), which also possess a strong oxidant ability to decolorize the MB molecules. In order to suppress the electron–hole pair recombination, H_2_O_2_, as an electron acceptor, would be added to the photocatalytic system to generate more ·OH radicals (eqn (6)). In the case of photocatalytic degradation of organic pollutants, the dye pollutants can be degraded by ·OH generated on the VB (eqn (7)), or directly oxidized by photo-excited h^+^ in the CB (eqn (8)).^32,33^

2 MIL-125 + *ℏυ* → h^+^ + e^−^

3 NH_2_-MIL-125 + *ℏυ* → h^+^ + e^−^

4 h^+^ + OH^−^ → ·OH

5 e^−^ + O_2_ → ·O_2_^−^

6 H_2_O_2_ + e^−^ → ·OH + OH^−^

7 Dye + ·OH → Degraded product

8 Dye + h^+^ → Degraded product

**Fig. 2:**
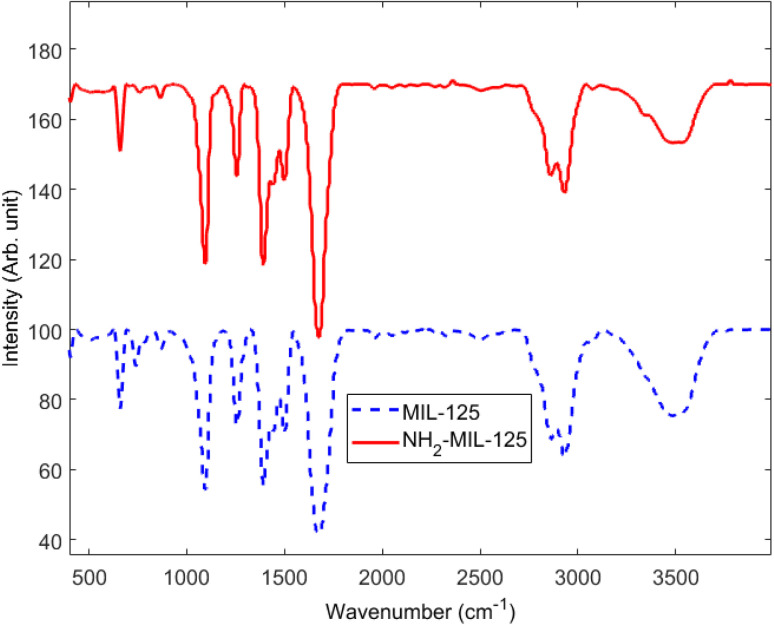
FTIR spectra of MIL-125 and NH_2_-MIL-125.

The FTIR spectra of MOFs are depicted in Fig. 2. In this work, we use MOFs in a methanol solution for FTIR analysis. The wide band at 3400 cm^−1^ can be attributed to O–H stretching vibration from the absorbed water molecules.^34^ The absorption bands ∼2900 cm^−1^ arise from C–H bond stretching vibrations. The acetyl group was observed by the C–O stretch at 1260 cm^−1^, while the C–O–C stretch, which represents the cellulose backbone, was observed at 1090 cm^−1^.^35^ In the case of NH_2_-MIL-125, the band between 3200 and 3500 cm^−1^ is wider than that of MIL-125, probably due to the stretch vibration of the –NH_2_ group at 3400 cm^−1^, overlapped with the characteristic band of adsorbed water.^36^ The R–COOH functional groups in the initial ligands underwent significant transformations upon complexation with Ti, resulting in the development of MIL-125 and NH_2_-MIL-125 solid. Notably, the disappearance of the carbonyl group (C=O) band at 1676 cm^−1^ for MIL-125 and 1678 cm^−1^ for NH_2_-MIL-125 and the emergence of two distinct bands at around 1500 cm^−1^ and 1350–1450 cm^−1^ could be appreciated, corresponding to asymmetrical and symmetrical stretching of the carboxylate group (COO^−^), respectively.^37^ All samples showed a doublet band from 500 to 800 cm^−1^, which could be attributed to the Ti–O vibration and a TiO oxo cluster of the MOF (there is a peak at 740 cm^−1^ in MIL-125 which disappeared in NH_2_-MIL-125).^38^ This indicates the induction of a resonance structure in the carboxylate group (–COO) through the interaction between Ti and the –COOH functional group of the ligands. These results provide evidence for the successful complexation and formation of titanium MOFs.

**Fig. 3:**
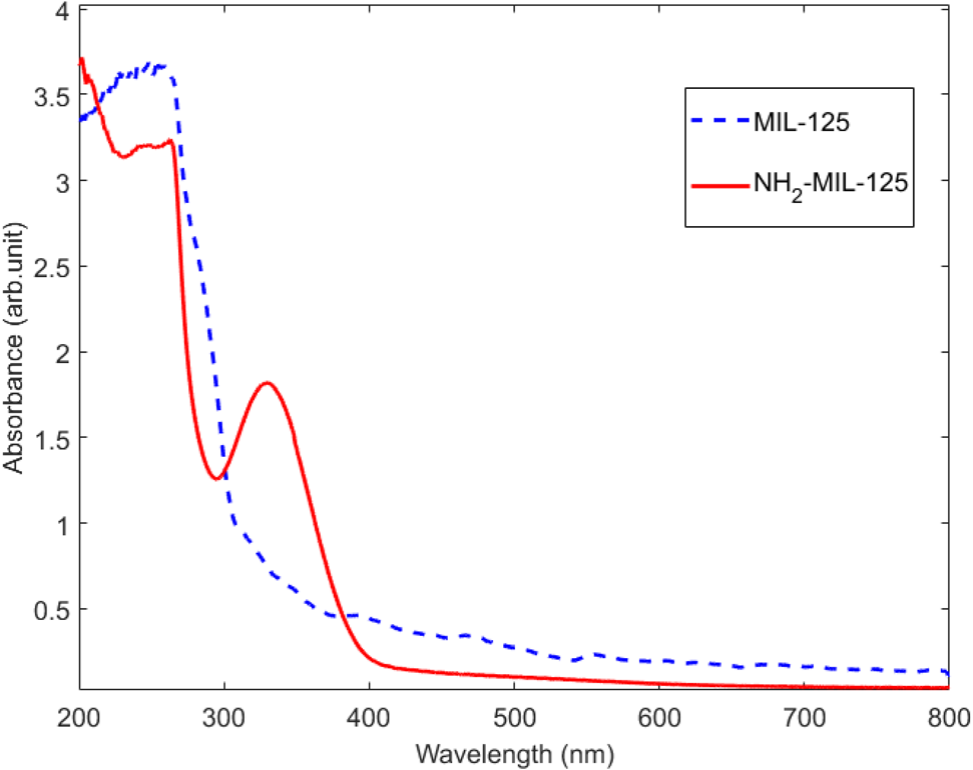
UV-visible spectra of MIL-125 and NH_2_-MIL-125.

UV-visible analysis depicted in Fig. 3 explored the optical absorption characteristics of pure MIL-125 and NH_2_-MIL-125. MIL-125 exhibits absorption peaks in the UVC and UVB range, while NH_2_-MIL-125 absorbs light across the UVC, UVB, UVA, and visible-light ranges, enhancing solar energy utilization and electron–hole pair production. MIL-125 primarily absorbs light below 347 nm, resulting in hole generation near the Ti-oxo cluster through excitation from the highest occupied crystal orbital (HOCO) to the lowest unoccupied crystal orbital (LUCO) defined by Ti(3d).^39–41^ Conversely, NH_2_-MIL-125 displays two absorption peaks: one below 300 nm, similar to MIL-125, and a second peak centered at 355 nm, indicating a ligand-to-metal cluster charge transfer (LMCT) mechanism. This involves excitation from the HOCO dominated by N from the ligand to the LUCO defined by Ti(3d), resulting in hole formation spatially separated in the ligand.^42,43^

**Fig. 4:**
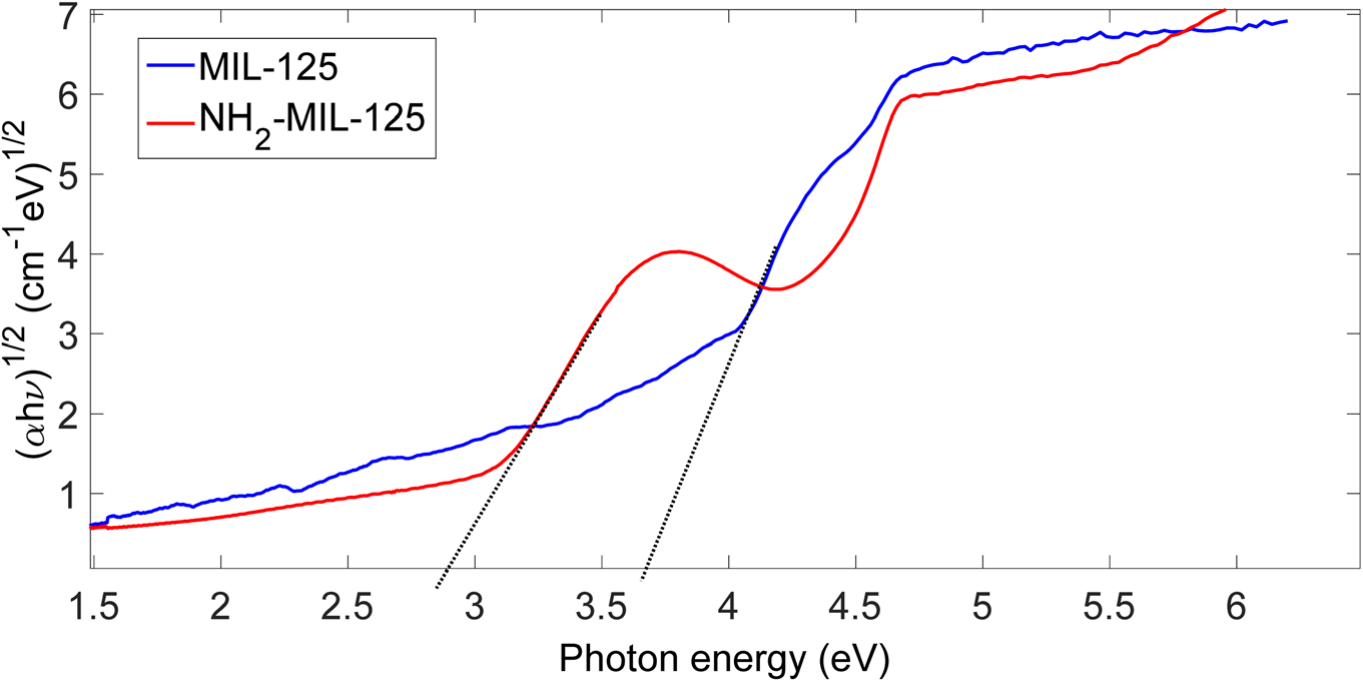
Bandgap (plots of (*αℏn*)^1/2^*versus* the photon energy) of MIL-125 and NH_2_-MIL-125.

Fig. 4 presents the determination of bandgap values using a Tauc plot. The Tauc plot, introduced by Tauc in 1966, calculates the bandgap energy of semiconductor materials from absorbance spectrum data.^44^ The Tauc equation for semiconductor material is given by:

9 (*α*·*ℏν*)^1/*n*^ = *B*(*ℏν* − *E*_g_)

Where *ℏ* is Planck's constant, *ν* is photon frequency, *E*_g_ is the bandgap energy, *B* is a constant, *n* is *a* factor depending on electron transition property, and *α* is the absorbance coefficient. The absorbance coefficient is calculated by:

10 *α* = ln(10) × *A*/*l*

Where *A* is absorbance, and *l* is the material thickness in centimeters.

Bandgap energy determination using a Tauc plot involves plotting (*αℏυ*)^(1/*n*)^ against *ℏυ* and extrapolating in the linear area across the energy axis in the corresponding graph. Consequently, the bandgap for MIL-125 is approximately 3.65 eV, while for NH_2_-MIL-125, it is 2.8 eV. Incorporating amino groups into the terephthalic acid (BDC) linker reduces the bandgap of MIL-125, indicating improved optical absorption properties. This smaller gap allows the photocatalyst with amino-functionalized linkers to absorb light more efficiently, enhancing its photocatalytic activity by trapping and sustaining photo-generated charges.

**Fig. 5:**
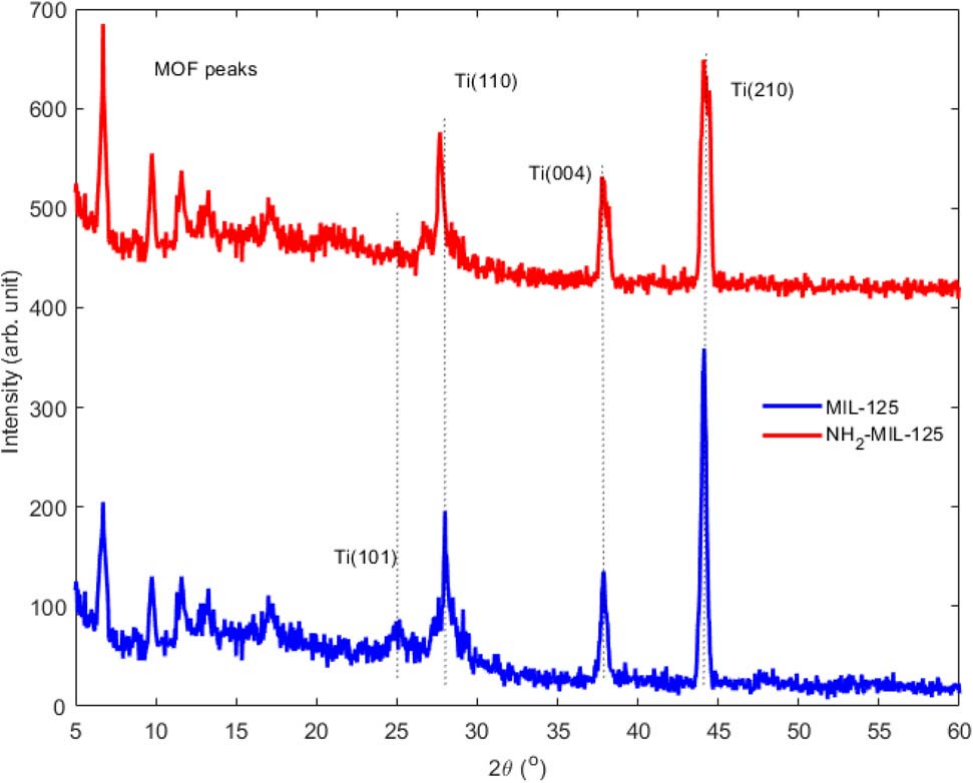
XRD of MIL-125 and NH_2_-MIL-125.

The XRD analysis of the as-prepared MOF (Fig. 5) reveals high crystallinity for both single-ligand MOFs, consistent with the crystal structures reported for MIL-125 and NH_2_-MIL-125.^45,46^ The presence of strong and sharp diffraction peaks indicates excellent sample crystallinity. The diffraction patterns of MIL-125 and NH_2_-MIL-125 exhibit significant similarity, suggesting that substituting –H with –NH_2_ in the ligand backbone has minimal impact on the framework structure. Notably, no additional peaks associated with other phases are observed in the diffraction patterns, confirming the purity of the prepared materials. Distinct diffraction peaks are observed at 2*θ* values of 6.72°, 9.72°, and 11.62°, corresponding to the (011), (020), and (121) planes,^47^ and at 25.08°, 27.6°, 37.76°, and 44.19°, indexed to the (101), (110), (004), and (210) planes as per JCPDS card no. 29-1360. Notably, the smaller crystal size of NH_2_-MIL-125 is evident from the wider and shorter diffraction peaks observed in most cases compared to MIL-125.

PL spectra serve as valuable tools for analyzing the efficiency of photo-generated electron–hole recombination. The PL samples, containing DMF and methanol along with MOF powder, were prepared before washing the MOFs. Various excitation wavelengths were investigated, with data obtained at 345 nm and 429 nm for MIL-125 and NH_2_-MIL-125, respectively, representing excitation within the BDC ligand and NH_2_ BDC ligand.

**Fig. 6:**
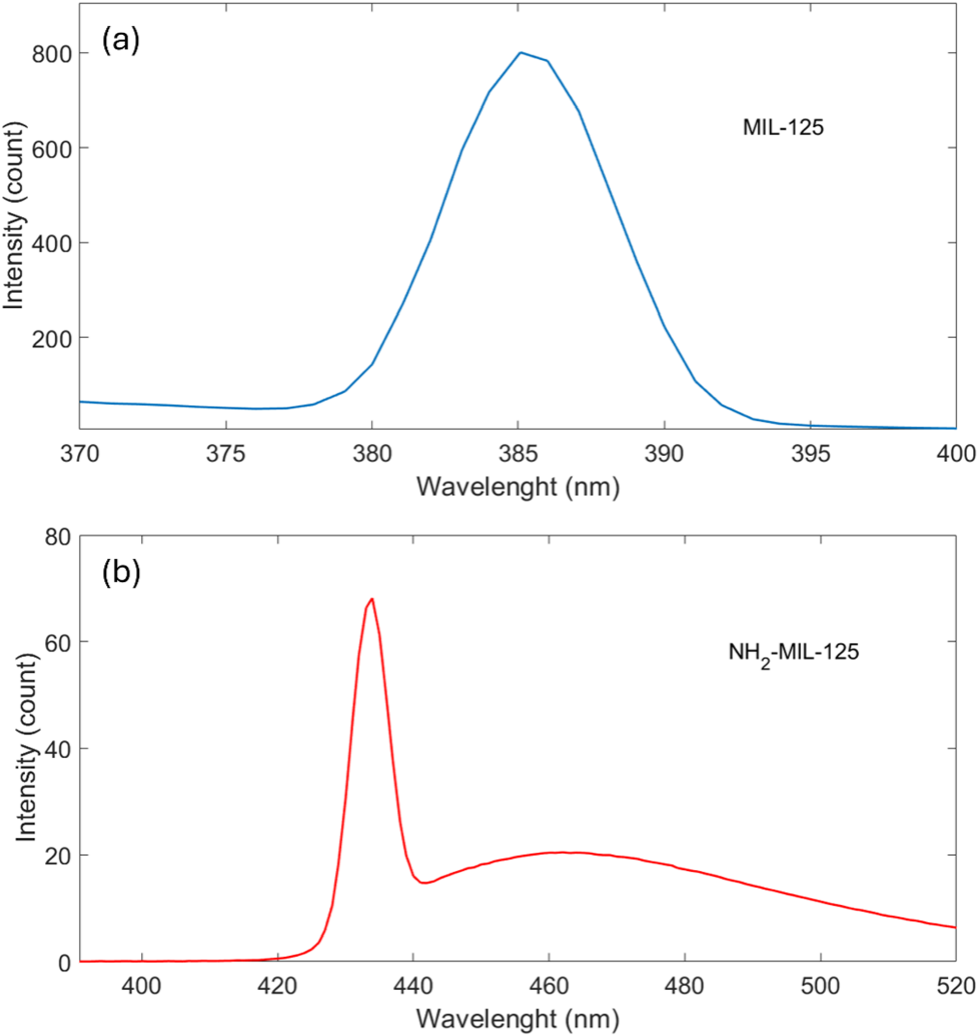
PL spectra of (a) MIL-125 and (b) NH_2_-MIL-125 nanostructures excited by high-intensity 345 nm and 429 nm beams, respectively.

In Fig. 6a, the corresponding emission spectra reveal a broad and strong emission peak centered at 385 nm. The lower emission intensity in solid-state PL can be attributed to differences in absorption efficiency and local concentrations between powders and suspensions. Fig. 6b presents the corresponding emission spectra of NH_2_-MIL-125, featuring a broad and strong emission peak centered at 433 nm. The emitted peaks at 433 nm could arise from intra-ligand recombination without involving the ligand-to-metal charge transfer excitation mechanism. Consequently, the emission originates from electron recombination, excited *via* ligand-to-metal charge transfer into the Ti(3d) orbitals, with holes residing at the N in the ligand.^48,49^

**Fig. 7:**
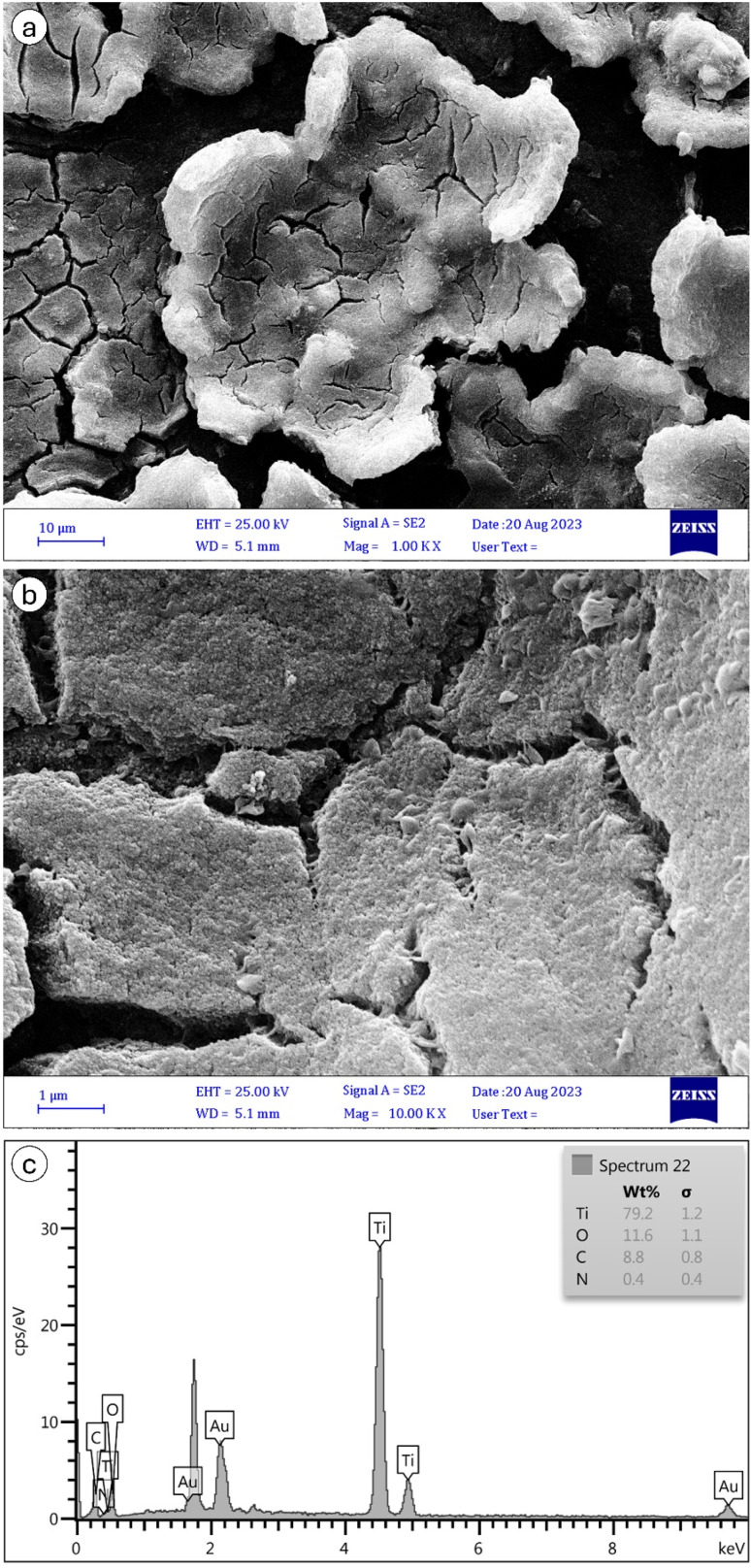
SEM images of MIL-125 nanostructures of (a) ×100 k and (b) ×10 k magnifications, and (c) its EDAX spectrum.

**Fig. 8:**
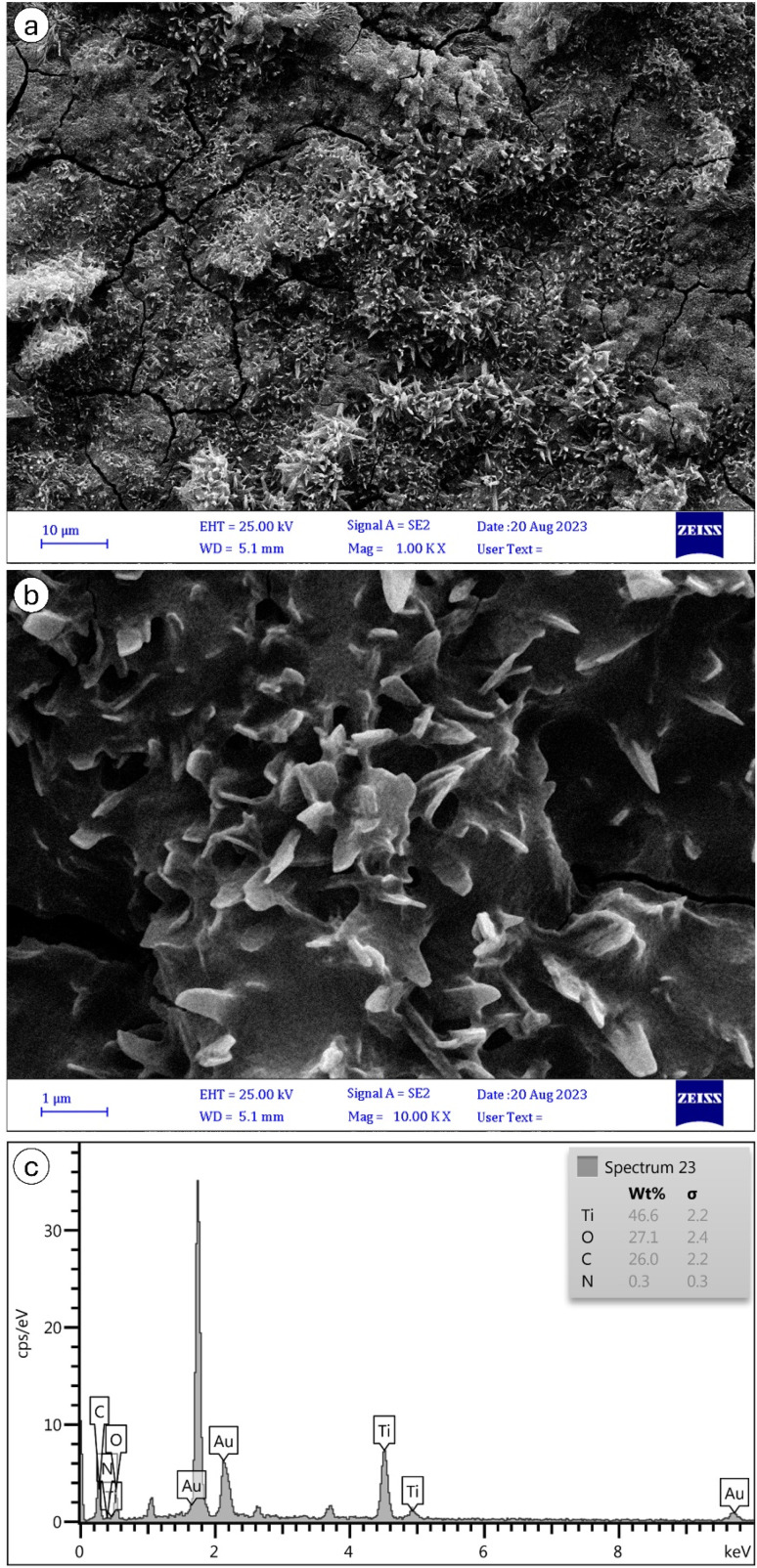
SEM images of NH_2_-MIL-125 nanostructures of (a) ×100 k and (b) ×10 k magnifications, and (c) its EDAX spectrum.

The FESEM characterization of MIL-125 and NH_2_-MIL-125, depicted in Fig. 7 and 8, respectively, provide insights into their morphological and structural details. The collected samples consist of well-crystallized block-like particles of varied forms, as observed in the low-magnification FESEM images. MIL-125 exhibits an elongated circular shape with a smooth surface. A closer examination of the surface reveals a rugged texture with large crevices (Fig. 7b). In contrast, Fig. 8b highlights aggregated quasi-spherical particles of NH_2_-MIL-125, resembling clusters. Further scrutiny of the NH_2_-MIL-125 surface reveals elongated quasi-spherical particles. All samples aggregate into three-dimensional spheres. MIL-125 possesses an average particle size of 40 nm. NH_2_-MIL-125 shares a similar shape with MIL-125 but exhibits a significantly smaller diameter or thickness, consistent with XRD pattern results (Fig. 5). The smaller crystal size corresponds to wider and shorter diffraction peaks. The elemental compositions of MIL-125 and NH_2_-MIL-125, determined by EDAX analysis, are depicted in Fig. 7c and 8c, respectively. These images indicate 79.2% Ti, 11.6% O, 8.8% C, and 0.4% N for MIL-125 and 46.6% Ti, 27.1% O, 26% C, and 0.3% N for NH_2_-MIL-125, which corresponds with the result of the FTIR (Fig. 2), because some peaks related to Ti, disappeared in NH_2_-MIL-125 indicating that the percentage of Ti in NH_2_-MIL-125 is fewer than Ti in MIL-125.

### 3.2. Photocatalytic activities

The absorption spectra of the MB reveal the most intense absorption peak at around 664 nm associated with an MB monomer, with a shoulder peak at about 612 nm attributed to an MB dimer. Two additional bands appear in the ultraviolet region with peaks around 292 and 245 nm (associated with substituted benzene rings).^50^ These absorption peaks gradually decrease as the photodegradation reaction proceeds.^51^ In this work, because of the UV-visible absorption of MOFs (Fig. 3), the MB's peak around 292 and 245 nm disappeared immediately after the experiment. In fact, the MB's peaks were hidden under the MOFs' peaks. Degradation was examined in darkness, under the UVC and visible light irradiation. Results are analyzed in detail in the following.

### 3.3. Darkness

**Fig. 9:**
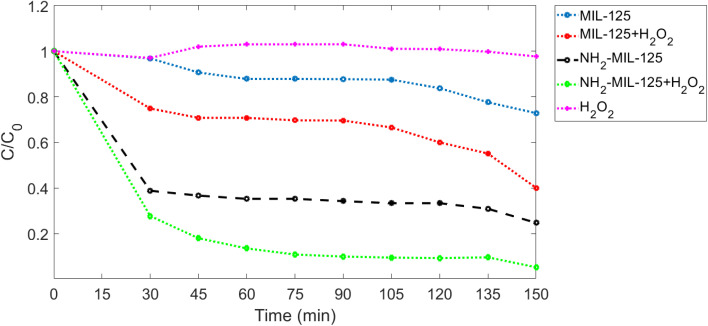
The MB degradation with different photocatalytic conditions in darkness.

The main results calculated from photocatalytic tests in darkness are shown in Fig. 9. The degradation of MB was negligible in darkness in the absence of photocatalyst MIL-125 or NH_2_-MIL-125, whereas the concentration of MB obviously decreased with taking time in the presence of a photocatalyst. The effect of NH_2_-MIL-125 on the degradation of MB is larger than MIL-125. This can be explained by several reasons. The existence of an amino group in the NH_2_-MIL-125 framework has greatly improved the adsorption properties of the material towards MB. The amino functional group in the NH_2_-MIL-125 framework contributes higher surface electronegativity due to the existence of lone electron pair in nitrogen, which attracts cationic MB, and this result correlated well with the study of Fan *et al.*,^52^ who found that the amino group in the NH_2_-MIL-125 also facilitates the formation of hydrogen bonding with MB in water which results in a stronger interaction force and a higher adsorption capacity than that of MIL-125.

Bandgaps can be effective, too; bandgaps are shown in Fig. 4. We considered that the bandgap of NH_2_-MIL-125 is smaller than MIL-125 because, with a smaller bandgap, less energy is required to promote electrons from the valence band to the conduction band. In the darkness, when there is no light available to generate electron–hole pairs, the bandgap is still relevant for photocatalysis because it influences the recombination rate of the electron–hole pairs. A narrower bandgap typically results in a higher recombination rate, meaning that the electron and hole are more likely to recombine and release their energy as heat rather than participate in a catalytic reaction. Conversely, a wider bandgap reduces the recombination rate, increasing the likelihood that the electron and hole will migrate to the surface of the material and participate in catalytic reactions even in the absence of light. This phenomenon is known as ‘dark catalysis’ and can occur when the bandgap is wide enough to allow the thermal excitation of electrons into the conduction band. Therefore, while light is necessary to initially generate electron–hole pairs in photocatalysis, the bandgap still plays a crucial role in determining the efficiency of catalytic reactions in darkness by influencing the recombination rate and the possibility of dark catalysis.

Fig. 9 shows the removal of MB for 150 minutes. In the dark environment, there are no photons that can activate the electrons. It leads to the dominance of adsorption in the dye degradation process, which results in an adsorption equilibrium. In a dark environment, the system reaches an absorption equilibrium where the rate of adsorption becomes equal to that of desorption. In this phase, organic pollutants from the surrounding environment adsorb onto the surface of the photocatalyst material. This adsorption process continues until the surface sites on the photocatalyst become saturated with adsorbate molecules, at which point the adsorption rate slows down and eventually reaches equilibrium. At adsorption equilibrium, the number of molecules desorbing from the surface of the photocatalyst equals the number of molecules adsorbing onto it, resulting in a stable concentration of adsorbate molecules on the photocatalyst surface. This equilibrium state sets the stage for subsequent reactions to occur when light is introduced to activate the photocatalyst. According to Fig. 9, in the first 30 minutes of the adsorption process, dye molecules have a high tendency to be adsorbed onto the membrane's surface; however, after a while, this tendency decreases, and the dye degradation rate becomes almost stable.^10^ In the first 30 minutes in darkness, it was observed that the concentration of MB solutions of NH_2_-MIL-125 was reduced by about 61% of initial MB solutions, while MIL-125 showed negligible adsorption capability of MB. It should be attributed to the high surface area, particle size and crystal structures of NH_2_-MIL-125. In general, particles with higher surface area, smaller size, and highly crystalline structures can provide more active sites for MB adsorption, which is consistent with the results obtained from PL (Fig. 6), SEM (Fig. 7 and 8), and XRD (Fig. 5).

In the absence of light, conventional photocatalytic reactions are halted as the essential light-driven electron transfer processes cannot occur. However, even in darkness, MIL-125 and NH_2_-MIL-125 can still exhibit some level of catalytic activity due to their inherent chemical properties. Studies have shown that MIL-125 and NH_2_-MIL-125 can adsorb MB molecules onto their surface through electrostatic interactions, leading to partial degradation of MB *via* non-photochemical pathways. While this process is significantly slower compared to photocatalysis under light irradiation, it highlights the unique adsorption and catalytic capabilities of MIL-125 and NH_2_-MIL-125, even in the absence of light.

As shown in Fig. 9, the photodegradation efficiencies of MB are both higher after adding H_2_O_2_. When the H_2_O_2_ and photocatalyst MIL-125 or NH_2_-MIL-125 were added into the solution together, the photocatalytic efficiency was notably improved due to the electron acceptor H_2_O_2_ that could suppress the electron–hole pair recombination, thus enhancing the photodegradation efficiency. For MIL-125, about 27.16% degradation of MB was observed after 150 min in darkness, while 59.93% MB degradation was achieved by adding H_2_O_2_. For NH_2_-MIL-125, about 75.28% degradation of MB was observed, while 94.75% MB degradation was achieved by adding H_2_O_2_.

The efficiency of MIL-125 in degrading methylene blue (MB) in darkness is primarily due to mechanisms such as dark catalysis and adsorption. MIL-125 has a porous structure and high surface area facilitate the adsorption of MB molecules, enhancing its degradation capability. Similarly, NH_2_-MIL-125 operates under the same mechanisms but is more effective due to its enhanced adsorption properties. The presence of amino groups in NH_2_-MIL-125 not only increases the surface area but also enhances surface electroactivity, leading to improved interaction with MB molecules.

Overall, NH_2_-MIL-125 has a comparative predominance for photocatalysis over MIL-125 in darkness. The kinetic constants (*k*) for MB photodegradation in darkness are 0.00135 min^−1^, 0.00432 min^−1^, 0.00664 min^−1^, 0.011 min^−1^ and 0.00074 min^−1^ for MIL-125, NH_2_-MIL-125, MIL-125 with H_2_O_2_, NH_2_-MIL-125 with H_2_O_2_ and H_2_O_2_ without photocatalyst, respectively. The result indicated the addition of an H_2_O_2_ electron acceptor could easily enhance the photocatalytic activities. The kinetic constant of MIL-125 is smaller than NH_2_-MIL-125. It was noticed that the crystallite size of MIL-125 is bigger than NH_2_-MIL-125 for the decrease of photocatalytic activity between them, and it is completely harmonized with SEM in Fig. 7 and 8.

### 3.4. UVC irradiation

**Fig. 10:**
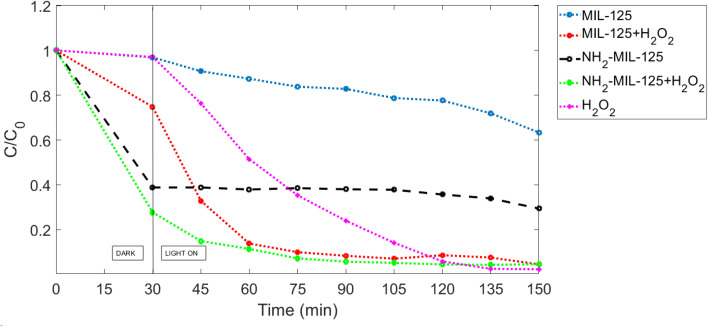
The MB degradation with different photocatalytic conditions in UVC.

When subjected to UVC lamp irradiation, MIL-125 and NH_2_-MIL-125 undergo photoexcitation, resulting in the generation of electron–hole pairs. These charged species then interact with adsorbed MB molecules, initiating a cascade of redox reactions that ultimately lead to the degradation of MB into smaller, less toxic by-products. The high energy of UVC light enables efficient activation of MIL-125 and NH_2_-MIL-125, making it a potent photocatalyst for the degradation of MB and other organic pollutants. Fig. 10 shows the removal curve of MB using various catalysts under UVC irradiation.

The degradation of MB was very high in UVC irradiation, unlike in darkness in the absence of photocatalyst MIL-125 or NH_2_-MIL-125. The data in UVC irradiation is different from the MB photocatalyst in darkness because, in darkness, the degradation in the absence of the photocatalyst was negligible. It is shown that in the first 30 min (in darkness), the photocatalyst was negligible, but after the light was switched on, it increased incredibly. H_2_O_2_ itself does not possess inherent photocatalytic activity in the traditional sense, as it does not undergo photoexcitation to generate electron–hole pairs like semiconductor photocatalysts such as titanium dioxide (TiO_2_). However, in the presence of UVC irradiation, H_2_O_2_ can still participate in the degradation of MB through non-photocatalytic pathways.

UVC irradiation is known to induce photolysis of H_2_O_2_, leading to the formation of hydroxyl radicals (·OH) and superoxide radicals (·O_2_^−^), which are highly reactive species capable of initiating the degradation of organic pollutants like MB. The chemical reactions involved in this process can be summarized as follows:

(1) Photolysis of H_2_O_2_:

11 H_2_O_2_ + UVC → ·OH + H_2_O

(2) Reaction with MB:

12 MB + ·OH → Degradation products

The hydroxyl radicals (·OH) generated through the photolysis of H_2_O_2_ react with MB, leading to its degradation into smaller, less harmful by-products. While this process does not involve a traditional photocatalyst material like TiO_2_, it still utilizes the reactive species generated by UVC irradiation of H_2_O_2_ to initiate the degradation of MB. Therefore, although H_2_O_2_ itself is not a photocatalyst, it can still facilitate the degradation of organic pollutants under certain conditions, such as UVC irradiation.

As can be seen from Fig. 10, before the light irradiation, MIL-125, NH_2_-MIL-125, MIL-125 + H_2_O_2_ and NH_2_-MIL-125 + H_2_O_2_ removed 3.15%, 61.2%, 25.13% and 72.3% of MB due to the adsorption. Under the irradiation of the UVC spectrum, NH_2_-MIL-125 displayed a larger MB removal (70.62%) than MIL-125 (36.68%). On the other hand, MIL-125 + H_2_O_2_ and NH_2_-MIL-125 + H_2_O_2_ displayed almost the same results (about 95%). NH_2_-MIL-125 exhibited better photocatalytic activity under irradiation of UVC, similar to the darkness condition. It can be seen that the photodegradation of MB in the absence of H_2_O_2_ is much lower than in the presence of H_2_O_2_ under the same experimental conditions. In Fig. 10, When the H_2_O_2_ and photocatalyst MIL-125 or NH_2_-MIL-125 were added into the solution together, the photocatalytic efficiency was clearly improved due to the electron acceptor H_2_O_2_ that could suppress the electron–hole pair recombination, thus enhancing the photodegradation efficiency.

For MIL-125, about 36.68% degradation of MB was observed after 150 min in UVC, while 95.51% MB degradation was achieved by adding H_2_O_2_. For NH_2_-MIL-125, about 70.62% degradation of MB was observed, while 95.42% MB degradation was achieved by adding H_2_O_2_. Overall, NH_2_-MIL-125 worked better than MIL-125 in UVC irradiation.

As shown in Fig. 10, in the first 30 minutes, NH_2_-MIL-125 showed more efficient removal than MIL-125. In photocatalysis, the decomposition of organic molecules can be divided into three steps: pollutant adsorption, chemical reaction, and the desorption of water, carbon dioxide, and by-products.^53^ Of these steps, pollutant adsorption is an important step conducted in the dark phase. At this step, the adsorption and desorption processes take place alternately, leading to the change of *C*/*C*_0_ in the course of a 30 minutes reaction. In this step, the adsorption occurs due to the interaction between the surface functional groups of the photocatalyst and the chemical functional groups of the organic pollutants, which have some stages. In the first stage, organic pollutants in the form of molecules or dissolved species come into contact with the surface of the photocatalyst material. In the second stage, adsorption can occur through various mechanisms, including physisorption and chemisorption. In the third stage, the photocatalyst material may have surface functional groups such as hydroxyl groups (–OH), carboxyl groups (–COOH), or amino groups (–NH_2_), which can facilitate the adsorption of organic pollutants through chemical interaction that is why there was a little removal of MB when using MIL-125. In contrast, all samples of NH_2_-MIL-125 showed high removal efficiency. As shown, in the presence of H_2_O_2,_ the removal of MB was better in MOFs because of hydroxyl groups (–OH).

The photodegradation of MB by NH_2_-MIL-125 is faster than the photodegradation by MIL-125, which is due to the low recombination of the exciting charge carrier. This results in capturing the electron, not recombining quickly back to the valence band. This fact has been confirmed by PL measurements in Fig. 6; MIL-125 has a stronger PL peak, and NH_2_-MIL-125 shows a lower PL peak. These results indicate that NH_2_-MIL-125 composites own lower recombination of the photo-generated electrons and holes.

The degradation of MB under UVC illumination using MIL-125 and NH_2_-MIL-125 is primarily driven by the presence of high energy photons. UVC light provides photons with sufficient energy to excite electrons from the valence band to the conduction band, promoting efficient electron–hole separation. This results in enhanced ROS generation and rapid photodegradation of MB. The higher photon energy of UVC light, compared to visible light, makes it more effective in activating these photocatalysts for the degradation process.

The kinetic constants (*k*) for MB photodegradation in UVC are 0.00245 min^−1^, 0.02119 min^−1^, 0.00589 min^−1^, 0.01525 min^−1^ and 0.0281 min^−1^ for MIL-125, NH_2_-MIL-125, MIL-125 with H_2_O_2_, NH_2_-MIL-125 with H_2_O_2_ and H_2_O_2_ without photocatalyst, respectively. The result indicated the addition of an H_2_O_2_ electron acceptor can easily enhance the photocatalytic activities. The kinetic constant of MIL-125 is smaller than NH_2_-MIL-125 in darkness, which is harmonized with SEM in Fig. 7 and 8.

### 3.5. Visible irradiation

**Fig. 11:**
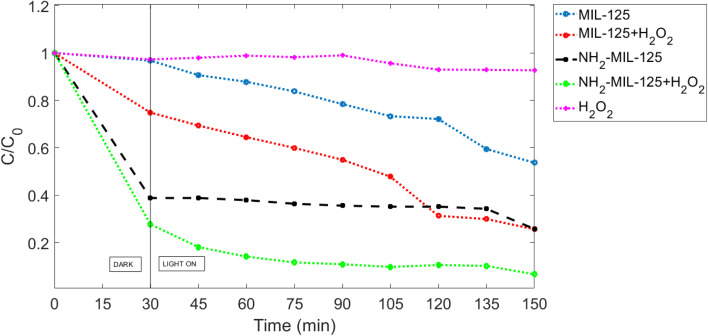
The MB degradation with different photocatalytic conditions in visible.

Visible light irradiation represents a more environmentally friendly approach to photocatalysis, utilizing solar energy for catalytic reactions. MIL-125 and NH_2_-MIL-125, with their bandgap energy tailored to absorb visible light, demonstrate remarkable photocatalytic activity under visible light illumination. When exposed to visible light, MIL-125 and NH_2_-MIL-125 harness photons to promote electron transfer processes, facilitating the degradation of MB molecules adsorbed on its surface. This environmentally benign approach to photocatalysis holds great promise for practical applications, offering a sustainable solution for the degradation of organic pollutants in aqueous environments. As depicted in Fig. 11, the degradation of MB with H_2_O_2_ was insignificant in the absence of photocatalysts MIL-125 or NH_2_-MIL-125 under visible light. However, in the presence of these photocatalysts, there was a noticeable decrease in the concentration of MB over time, while NH_2_-MIL-125 exhibited a significantly higher photodegradation rate compared to MIL-125 due to the existence of the amino group, higher surface area and slightly lower bandgap. This disparity could be attributed to the smaller bandgap of NH_2_-MIL-125, as illustrated in Fig. 4.

Furthermore, as shown in Fig. 11, the photodegradation efficiencies of MB were notably enhanced when H_2_O_2_ was added. H_2_O_2_ further improved the photocatalytic efficiency when combined with MIL-125 or NH_2_-MIL-125, as it acted as an electron acceptor, suppressing electron–hole pair recombination and thereby enhancing photodegradation efficiency. There was approximately 46.31% degradation of MB with MIL-125 and 74.27% degradation with NH_2_-MIL-125 in visible light. However, when H_2_O_2_ was added, the degradation percentages increased to 74.36% and 92.63% for MIL-125 and NH_2_-MIL-125, respectively. This difference is much clearer and more evident in the case of MIL-125 because according to Tauc law, the bandgap of MIL-125 is 3.65 eV, which is more compared to NH_2_-MIL-125, and this means it doesn't work very well as a photocatalyst in the absence of H_2_O_2_.

The effectiveness of MIL-125 in degrading MB under visible light can be attributed to several factors, including bandgap activation, electron–hole pair formation, ROS generation, and subsequent photodegradation. These mechanisms are also advantageous for NH_2_-MIL-125; the presence of amino groups adds an additional benefit through LMCT mechanisms. This process involves the excitation of electrons from the highest occupied molecular orbitals to the lowest unoccupied molecular orbitals, further enhancing the photocatalytic activity of NH_2_-MIL-125 under visible light.

The calculated kinetic constants (*k*) for MB photodegradation in visible light were 0.00288 min^−1^, 0.00901 min^−1^, 0.0062 min^−1^, 0.0098 min^−1^, and 0.00092 min^−1^ for MIL-125, NH_2_-MIL-125, MIL-125 with H_2_O_2_, NH_2_-MIL-125 with H_2_O_2_, and H_2_O_2_ without photocatalyst, respectively. These results indicate that adding the electron acceptor H_2_O_2_ significantly enhances photocatalytic activity, with NH_2_-MIL-125 demonstrating a higher kinetic constant than MIL-125. This observation is consistent with the SEM images in Fig. 7 and 8, which show that the crystallite size of MIL-125 is larger than that of NH_2_-MIL-125, contributing to the differences in photocatalytic activity between the two catalysts.

Adding H_2_O_2_ also has some disadvantages. H_2_O_2_ has poor UV light absorption characteristics. Thus, this can be considered as wasting most of the light input. In the Fenton process, producing sludge containing iron hydroxide as a by-product is a major drawback.^54^

**Fig. 12:**
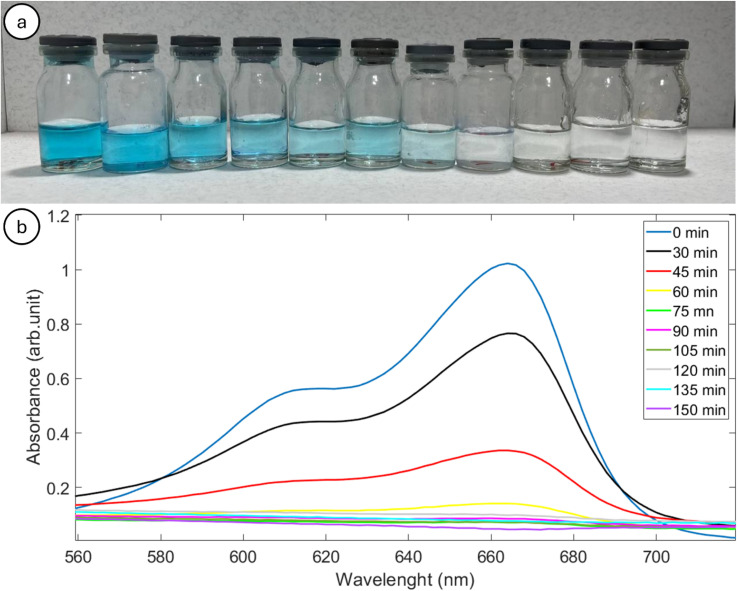
(a) Photograph of the vials containing MB at different irradiation times, from left to right: 0, 15, 30, 45, 60, 75, 90, 105, 120, 135 and 150 min. (b) Absorption spectra of MB with respect to irradiation time over MIL-125 and H_2_O_2_ in UVC irradiation. The light was turned on from 30 minutes onward.

The percentage of MB degradation is directly related to the irradiation time, which means degradation increases with increasing irradiation time.^11,55^Fig. 12a shows a color change from blue to colorless, and the reduction of MB chromophore is probably the reason for the decrease in absorption spectra. Fig. 12b presents the absorption spectra of MB under UVC irradiation for MIL-125 and H_2_O_2_. The other absorption spectra related to other conditions can be found in the supplementary Fig. S1 to S5.† Fig. S6† shows additional photographs from the vials in different conditions.

Shaban M. *et al.*^56^ indicated that under photocatalytic circumstances, the oxidation of MB to H_2_O and CO_2_ is an imperative technique to remove the dye from industrial wastewater. The MB dye solution's absorption peak was at 664 nm, and it gradually decreased due to dye degradation, eventually reaching its lowest value at 150 min. The photocatalyst cleavage of the aromatic ring of the dye molecules and the initiation of its degradation are thought to be the cause of the decrease in absorption peaks.^57^ The distinct absorption peak of MB spectra gradually decreases with the increase in reaction time.

## 4. Conclusion

The presence of MB in fresh water is harmful to humans and harms microbes and aquatic life due to its toxic nature. Photodegradation has emerged as a cost-effective method for fully decolorizing and mineralizing MB dye into harmless compounds. Ti-based MOFs are highly efficient photocatalysts in the degradation of water-solved MB molecules. They are easy and inexpensive to synthesize, and their performance under visible light irradiation is remarkable. In this work, MIL-125 and NH_2_-MIL-125 were successfully synthesized using the solvothermal method. NH_2_-MIL-125 typically refers to MIL-125 that has been functionalized with amino groups. The photocatalytic performance of MIL-125 and NH_2_-MIL-125 was tested for the degradation of MB in darkness and under the irradiation of visible light and UVC lamp. The results of MB photodegradation showed good photocatalytic performance of two materials. NH_2_-MIL-125 exhibited a significantly higher photodegradation rate compared to MIL-125 due to the existence of the amino group, higher surface area and slightly lower bandgap. This result correlated well with the study of Fan *et al.*^52^ Hydroxyl radicals (·OH) play a significant role in MB degradation; H_2_O_2_ increases the formation rate of hydroxyl radicals and enhances the degradation of compounds at low concentrations. Adding H_2_O_2_ can enhance the MB degradation efficiency by suppressing the recombination of photo-generated electron–hole pair, especially NH_2_-MIL-125, in all conditions. Furthermore, the effect of H_2_O_2_ as an electron acceptor on the efficiency of MB degradation was investigated, which markedly enhanced the photocatalytic MB degradation performance due to the ligand-to-metal charge transfer mechanism,^58–60^ particularly for NH_2_-MIL-125, under all tested conditions. In the presence of H_2_O_2_, there is only a minimal difference between the dye removal efficiency under visible light and UVC light irradiation, which motivates using the visible light due to the availability of this irradiation source.

The result reveals that the synthesized Ti-based MOF is an environmental catalyst for removing wastewater contaminants, especially when used with H_2_O_2_. This result made them a fine candidate for applications in environmental purification.

## Conflicts of interest

There are no conflicts to declare.

## Supplementary Material

RA-014-D4RA05733C-s001
