# Supplementary material for: Significant effects of negligible amount of H2O2 on photocatalytic efficiency of MIL-125 and NH2-MIL-125 nanostructures in degradation of methylene blue
Source: RSC Adv. 2024 Sep 23;14(41):30140–53. doi: 10.1039/d4ra05733c (PMC11418012; doi:10.1039/d4ra05733c)
Supplement: RA-014-D4RA05733C-s001 [file RA-014-D4RA05733C-s001.pdf]

This journal is © The Royal Society of Chemistry 2024

Supplementary figures S1–6.

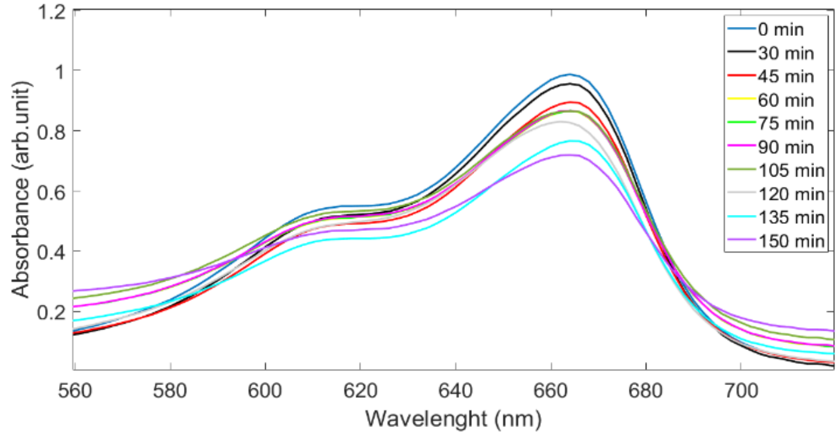

a) MIL-125 – Darkness

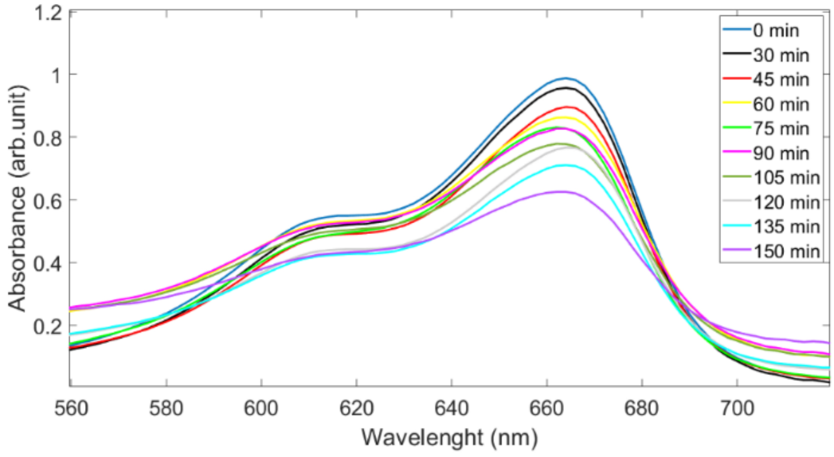

b) MIL-125 – UVC

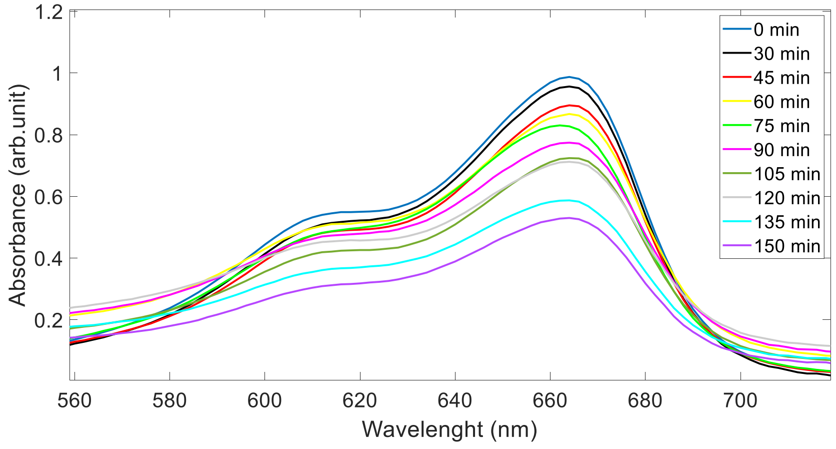

c) MIL-125 – Visible

Fig. S1. Absorption spectra of MB after adding MIL-125 in (a) darkness, (b) UVC, and (c) visible light irradiation. In (b) and (c), the light was turned on only after 30 minutes.

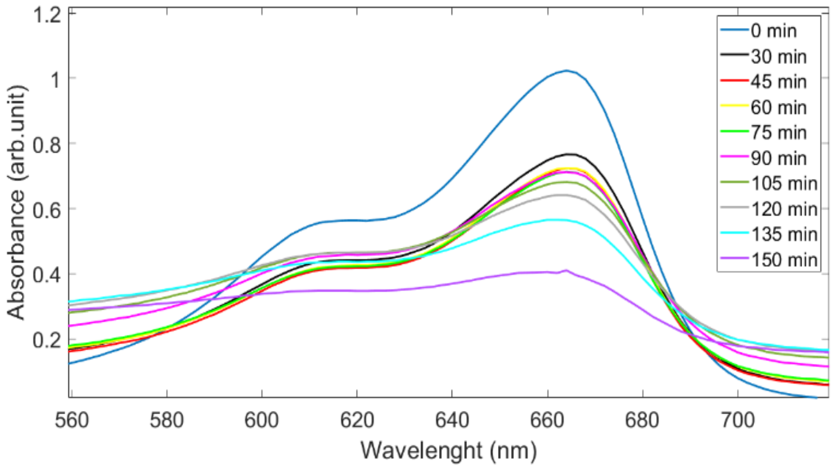

a) MIL-125+H_2O_2 – Darkness

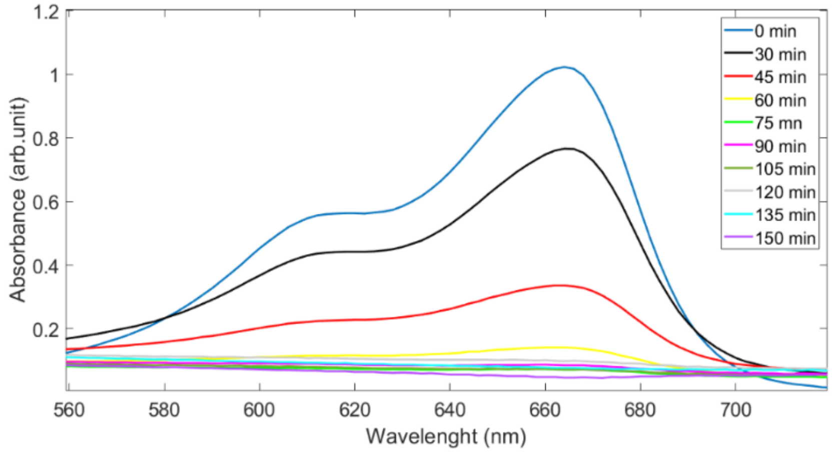

b) MIL-125+H_2O_2 – UVC

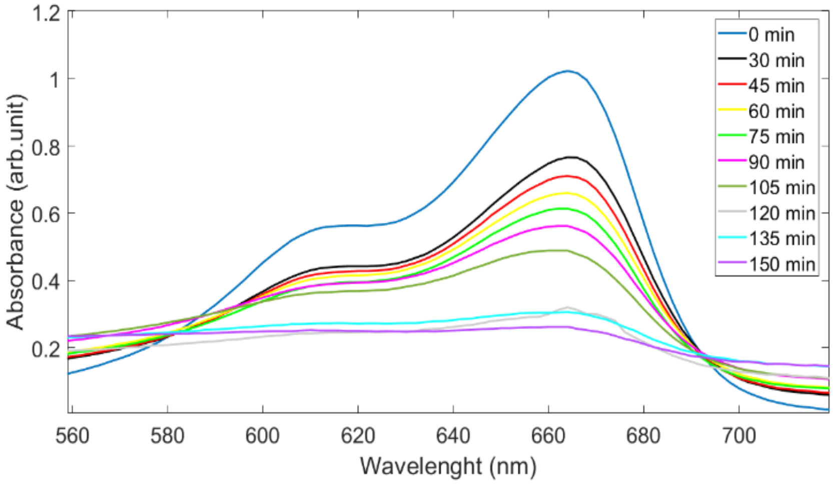

c) MIL-125+H_2O_2 – Visible

Fig. S2. Absorption spectra of MB after adding MIL-125+H_2O_2 in (a) darkness, (b) UVC, and (c) visible light irradiation. In (b) and (c), the light was turned on only after 30 minutes.

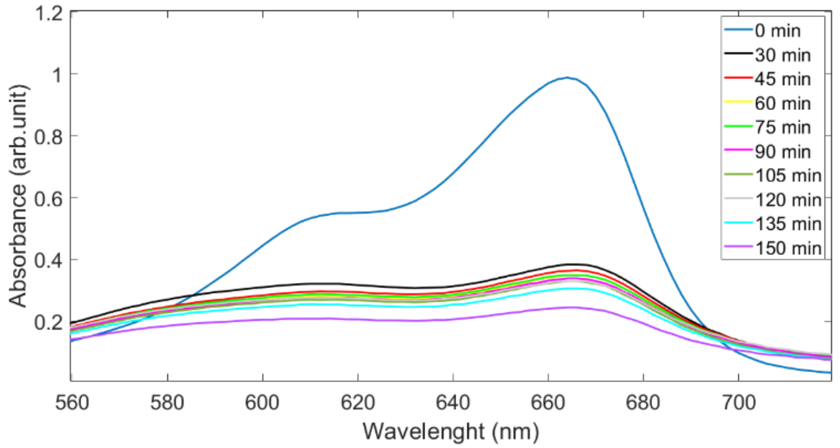

a) NH_2-MIL-125 – Darkness

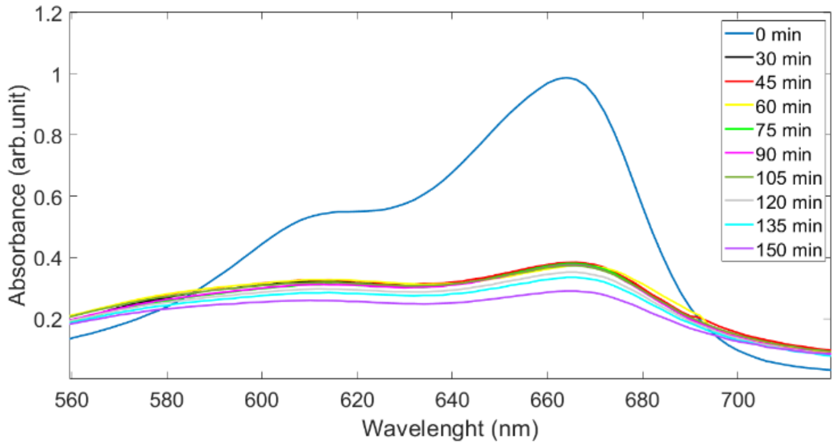

b) NH_2-MIL-125 – UVC

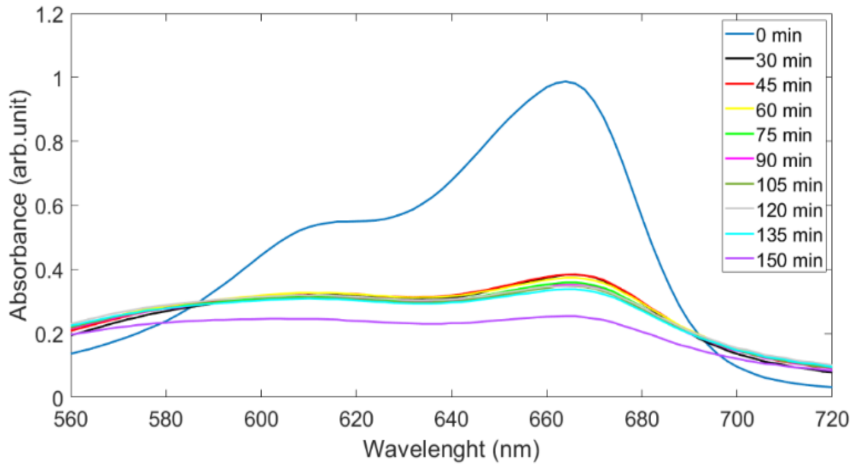

c) NH_2-MIL-125 – Visible

Fig. S3. Absorption spectra of MB after adding NH_2-MIL-125 in (a) darkness, (b) UVC, and (c) visible light irradiation. In (b) and (c), the light was turned on only after 30 minutes.

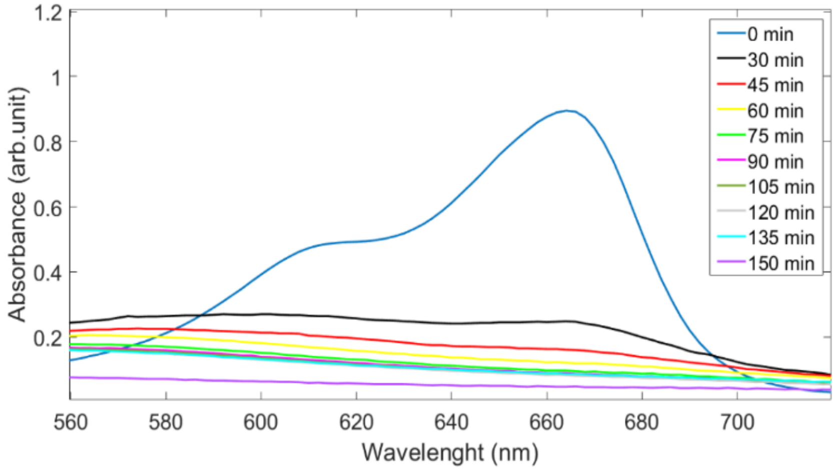

a) NH_2-MIL-125+H_2O_2 – Darkness

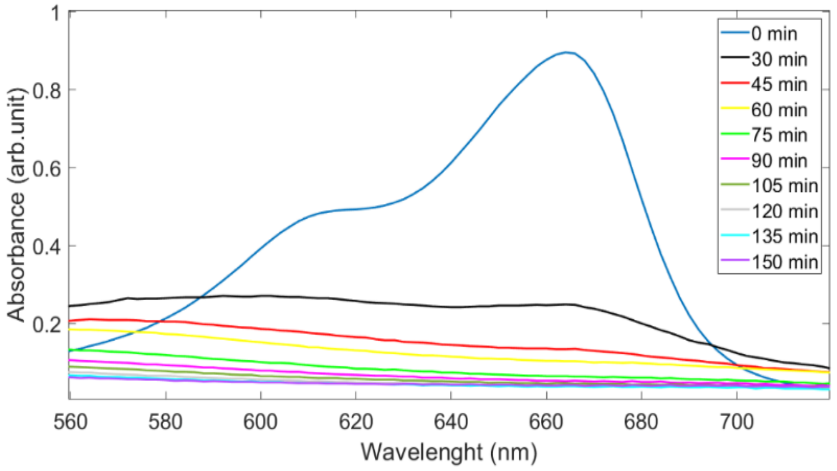

b) NH_2-MIL-125+H_2O_2 – UVC

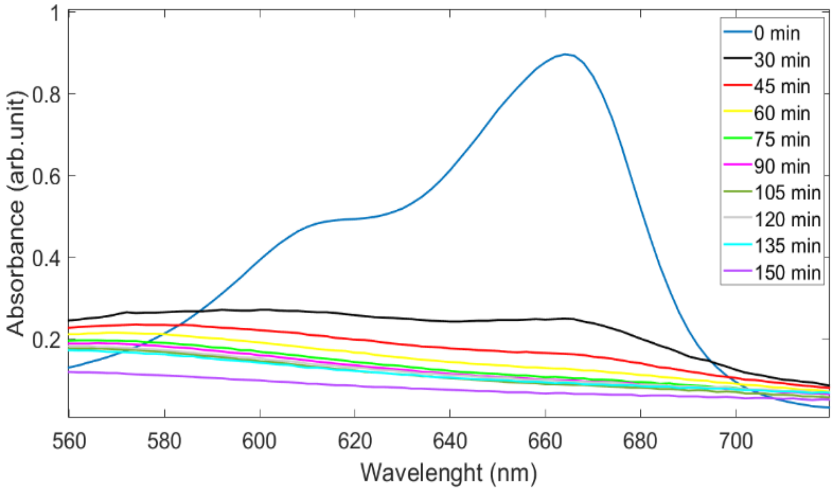

c) NH_2-MIL-125+H_2O_2 – Visible

Fig. S4. Absorption spectra of MB after adding NH_2-MIL-125+H_2O_2 in (a) darkness, (b) UVC, and (c) visible light irradiation. In (b) and (c), the light was turned on only after 30 minutes.

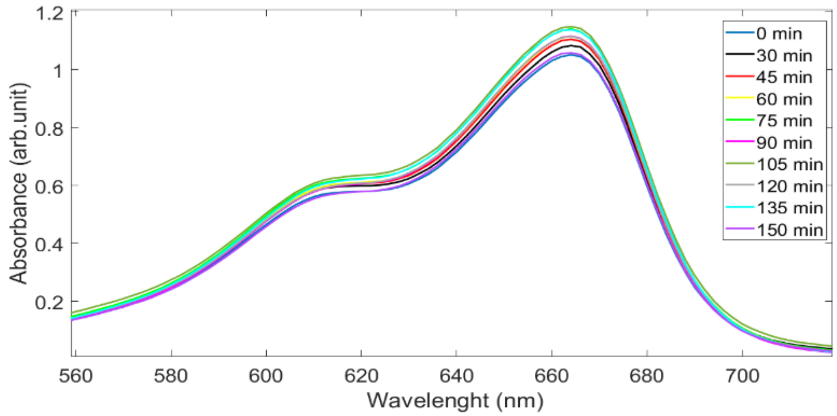

a) H_2O_2 – Darkness

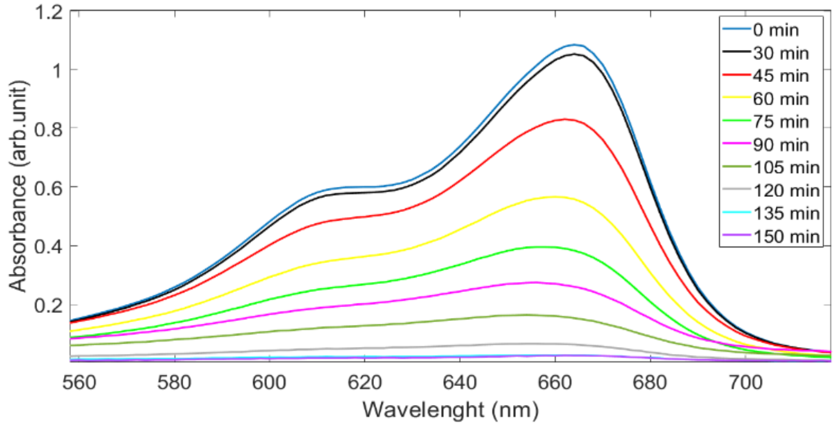

b) H_2O_2 – UVC

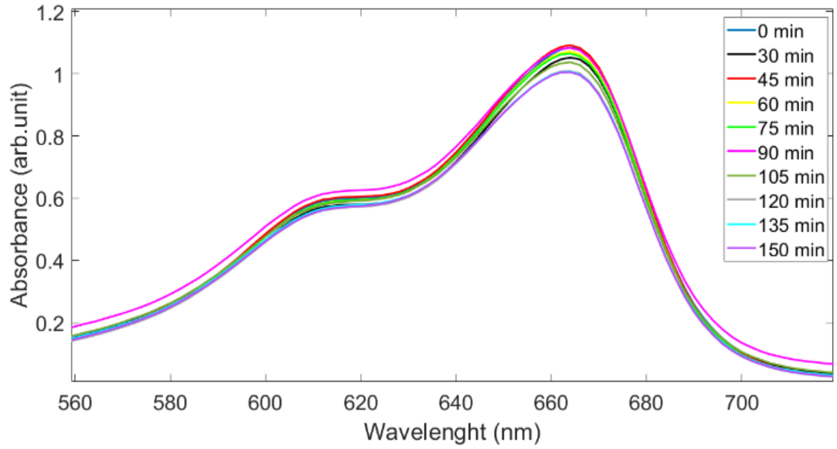

c) H_2O_2 – Visible

Fig. S5. Absorption spectra of MB after adding H_2O_2 in (a) darkness, (b) UVC, and (c) visible light irradiation. In (b) and (c), the light was turned on only after 30 minutes.

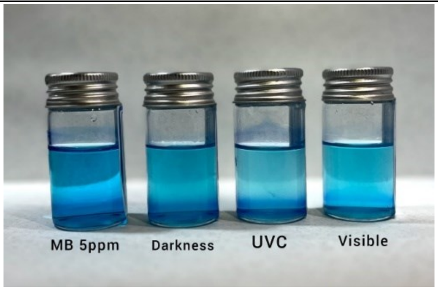

a) Photograph of the vials containing (from left to right): MB 5ppm (original solution), MB+MIL-125 after 150 min in darkness, under the UVC and visible light irradiation.

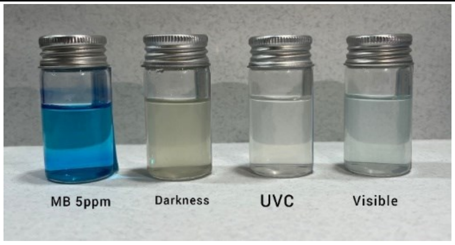

b) Photograph of the vials containing (from left to right): MB 5ppm (original solution), MB+MIL-125+H_2O_2 after 150 min in darkness, under the UVC and visible light irradiation.

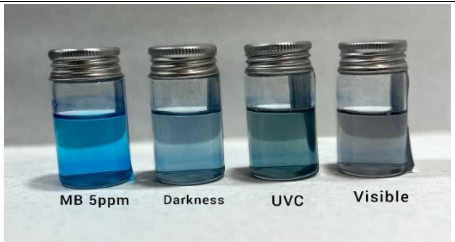

c) Photograph of the vials containing (from left to right): MB 5ppm (original solution), MB+NH_2-MIL-125 after 150 min in darkness, under the UVC and visible light irradiation.

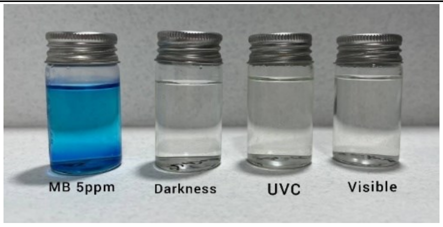

d) Photograph of the vials containing (from left to right): MB 5ppm (original solution), MB+NH_2-MIL-125+H_2O_2 after 150 min in darkness, under the UVC and visible light irradiation.

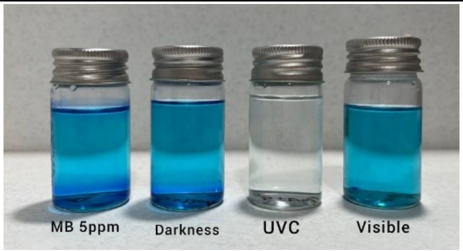

e) Photograph of the vials containing (from left to right): MB 5ppm (original solution), MB+H_2O_2 after 150 min in darkness, under the UVC and visible light irradiation.

Fig. S6. Photograph of the vials containing MB and (a) MIL-125, (b) MIL-125+H_2O_2, (c) MB+NH_2-MIL-125, (d) NH_2-MIL-125+H_2O_2, and (e) H_2O_2, after 150 min in darkness, under the UVC and under visible light irradiation.
